# Factors Associated With Having a Physician, Nurse Practitioner, or Physician Assistant as Primary Care Provider for Veterans With Diabetes Mellitus

**DOI:** 10.1177/0046958017712762

**Published:** 2017-06-15

**Authors:** Perri Morgan, Christine M. Everett, Valerie A. Smith, Sandra Woolson, David Edelman, Cristina C. Hendrix, Theodore S. Z. Berkowitz, Brandolyn White, George L. Jackson

**Affiliations:** 1 Duke University, Durham, NC, USA; 2 Durham Veterans Affairs Medical Center, Durham, NC, USA

**Keywords:** Veterans Administration, nurse practitioner, physician assistant, primary care, health workforce

## Abstract

Expanded use of nurse practitioners (NPs) and physician assistants (PAs) is a potential solution to workforce issues, but little is known about how NPs and PAs can best be used. Our study examines whether medical and social complexity of patients is associated with whether their primary care provider (PCP) type is a physician, NP, or PA. In this national retrospective cohort study, we use 2012-2013 national Veterans Administration (VA) electronic health record data from 374 223 veterans to examine whether PCP type is associated with patient, clinic, and state-level factors representing medical and social complexity, adjusting for all variables simultaneously using a generalized logit model. Results indicate that patients with physician PCPs are modestly more medically complex than those with NP or PA PCPs. For the group having a Diagnostic Cost Group (DCG) score >2.0 compared with the group having DCG <0.5, odds of having an NP or a PA were lower than for having a physician PCP (NP odds ratio [OR] = 0.83, 95% confidence interval [CI]: 0.79-0.88; PA OR = 0.85, CI: 0.80-0.89). Social complexity is not consistently associated with PCP type. Overall, we found minor differences in provider type assignment. This study improves on previous work by using a large national dataset that accurately ascribes the work of NPs and PAs, analyzing at the patient level, analyzing NPs and PAs separately, and addressing social as well as medical complexity. This is a requisite step toward studies that compare patient outcomes by provider type.

## Background

New approaches to caring for patients with diabetes are needed due to projected increases in diabetes prevalence, suboptimal quality of diabetes care, the heavy cost burden of diabetes, and expected insufficient numbers of primary care physicians in coming years. In 2012, 12% of the US adult population had diabetes, and this figure is expected to reach 20% to 33% by 2050.^1,2^ Most patients receive care for their diabetes in primary care settings,^3,4^ but estimates of primary care physician shortfalls range as high as 31 000 by 2025.^5^ Many patients with diabetes do not meet quality standards for routine preventive care^2^ or achieve recommended disease control targets.^6-8^ Meanwhile, complications such as heart disease, kidney failure, amputations, and loss of vision contribute to the human and financial costs of the disease.^9^ In 2012, direct medical costs in the United States for diabetes care were approximately $176 billion.^9^

Innovative approaches to primary care, such as patient-centered medical home (PCMH) models and expanded use of nurse practitioners (NPs) and physician assistants (PAs), are being implemented to address these access, quality, and cost problems.^10-15^ PCMH models are a multifaceted approach to primary care designed to better meet the needs of patients with chronic disease. Expanded use of PAs and NPs is a common approach to address workforce deficiencies, both within and outside of PCMH models, but research demonstrating the most effective utilization of these professionals in primary care is scant.^16^

The Veterans Health Administration (VHA) system is in the vanguard of implementation of both strategies discussed above: adopting a PCMH model and expanding use of NPs and PAs in primary care. It is the largest US employer of PAs and NPs and has been a pioneer in adopting expansive roles for NPs and PAs.^17-19^ The VHA’s version of the medical home approach, called the Patient-Aligned Care Team (PACT) model, has now been implemented nationwide.^14,20^ Each PACT is led by an NP, PA, or physician who is responsible for the medical care of a panel of veterans.^21,22^

Patient characteristics might lead to preferential assignment to a PACT led by a particular type of provider. For example, it is possible that the most medically complex patients might be assigned to physicians due to physicians’ advanced medical training, or that patients with significant psychosocial needs might be assigned to NPs due to the NP profession’s training emphasis on psychosocial aspects of well-being.^23,24^ Previous research in the VHA and in other settings regarding associations of provider type with patient demographic and medical complexity factors has produced mixed results,^16^ but a number of studies have found that patients seeing physicians are slightly older and slightly more medically complex and that NPs are substantially more likely to see female patients.^25-30^

Patient assignment to a particular provider type might also be affected by facility or state-level contextual factors. There are no standard criteria for assigning veterans to particular primary care provider (PCP) types. Wide variations in use of NPs and PAs across VHA facilities are well documented,^19,29^ including by our 2010 analysis showing that the proportion of primary care visits attended by PAs or NPs varied across regional Veterans Integrated Service Networks (VISNs) from 14% to 42%.^27^ As a number of studies have shown that PAs and NPs are more likely to provide care in rural areas than are physicians,^25,27,30,31^ patients attending rural clinics might be more likely to be assigned to an NP or PA. There is also variation in the use of PAs and NPs by region and state. In an analysis of National Ambulatory Health Care Survey data, the proportion of nonfederal primary care physicians who reported working with an NP or PA in 2012 varied from as low as 35% in Georgia to as high as 90% in South Dakota and was higher in practices with more physicians and in multispecialty practices.^32^ State scope of practice (SOP) regulations may also affect the degree to which PAs and NPs are used in the VHA even though federal supremacy rules grant NPs and PAs somewhat wider SOP in the VHA than in other settings. PAs and NPs are more concentrated in states with less restrictive SOP regulations, although the causal direction of this relationship is unclear.^33,34^ The increased availability of NPs and PAs in these states might facilitate their uptake in the VHA, thereby affecting patients’ likelihood of receiving care from them.

### New Contribution

This article analyzes patient, facility, and state characteristics associated with having a physician, NP, or PA as the VHA PCP for patients with diabetes, with a particular focus on the association between patient medical and social complexity and assigned provider type. Understanding factors associated with having a particular type of PCP is essential for primary care workforce planning. Although other studies have examined patient and facility characteristics associated with provider type, our study differs by being conducted within the largest integrated delivery system in the United States, which explicitly includes NPs and PAs as leaders of primary care teams. Unlike most other studies, our study examines continuous care of chronic disease over the course of 2 years and evaluates social complexity in addition to medical complexity and demographic factors.

Although the VHA population is predominantly male and on average older, sicker, and of lower socioeconomic status than the general population,^35,36^ our use of VHA data overcomes a number of obstacles to research comparing provider types. Most importantly, VHA electronic health record (EHR) data are the only longitudinal national data source that accurately ascribes care to PAs and NPs.^37^ In contrast, Medicare and Medicaid data are inaccurate for studying primary care PAs and NPs because their care is frequently subsumed under physician care due to billing practices (“incident to” billing).^37^ Use of a national data source was essential so that the effect of state-level factors, such as SOP regulations, could be examined.^38^ Finally, VHA data provide a broad range of variables for our examination and sufficient sample size (N = 377 579) to support statistical procedures.^39,40^

Examination of the similarities and differences of patients whose PCPs are physicians, NPs, or PAs is a requisite step toward conduct of studies comparing access, quality, and cost outcomes across provider types. Taken together, these studies may contribute to improvements in primary care of patients with diabetes.

## Methods

### Data Sources and Sample Construction

**Figure 1.**
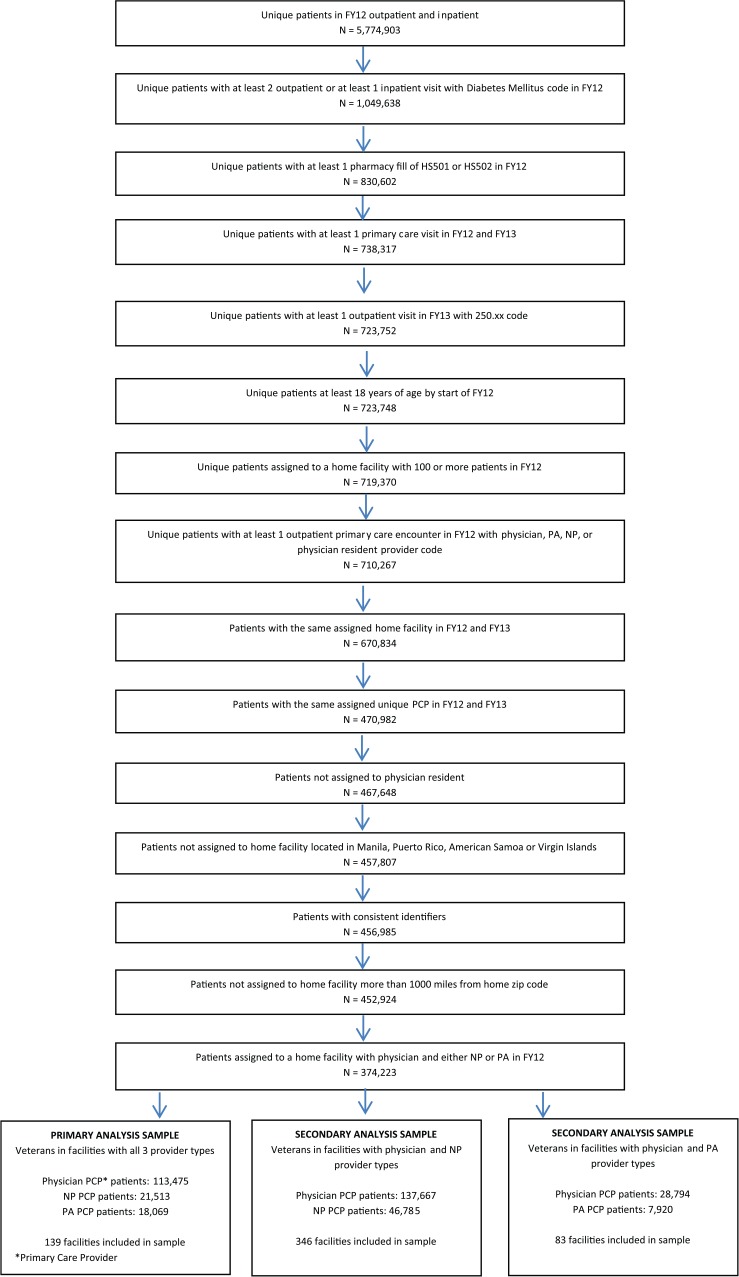
Primary care providers for veterans with diabetes mellitus cohort construction flow chart. *Note.* PA = physician assistant; NP = nurse practitioner; PCP = primary care provider.

This cohort study used centrally available national VHA EHR data. The construction of the cohort is summarized in Figure 1. Our national sample consisted of adult, pharmaceutically treated diabetes patients seen within VHA primary care clinics. Specifically, patients must have had a diabetes diagnosis (International Classification of Diseases, Ninth revision [ICD-9] codes 250.xx) associated with at least 1 VHA inpatient visit and/or at least 2 VHA outpatient visits in fiscal year (FY) 2012 (October 1, 2011, to September 30, 2012; N = 1 049 638) and a filled prescription for insulin and/or an oral hyperglycemic agent (VHA drug classes HS501 or HS502) the same year (N = 830 602). These individuals had to have at least 1 VHA primary care visit in FY 2012 using VHA administrative codes indicating a primary care clinic (VHA stop codes 322, 323, 342, and 348). Patients were excluded if they did not also have an outpatient visit with a diabetes diagnosis in FY 2013 or were younger than 18 at the beginning of FY 2012. Each patient was assigned a “home” facility as the clinic most frequently visited for primary care in FY 2012. To be retained in the cohort, patients had to have a “home” facility with at least 100 cohort members in FY 2012 (remaining N = 719 370). The provider most often visited in the home VHA’s primary care clinic in FY 2012 was considered to be the veteran’s PCP. The same procedure was used to determine home clinic and PCP in FY 2013. To ensure consistency in the patient-provider relationship, we excluded veterans whose PCP assignment changed between 2012 and 2013. As our primary interest was on factors associated with type of nontrainee provider seen by VHA diabetic patients, we excluded patients who most frequently saw a physician resident. We also excluded patients whose home facility (whether a traditional facility or a community-based outpatient clinic [CBOC]) was over 1000 miles from their home zip code or was not in one of the 50 U.S. states or the District of Columbia (remaining N = 456 985).

Our original planned analysis was to examine facilities in which all 3 provider types served as PCP for cohort patients. This remains our primary analysis. However, because only 139 of 823 facilities had patients with all 3 PCP types based on our algorithm of determining PCP, we decided to additionally examine associations within facilities that had only 2 PCP types as secondary analyses. Thus, in total, our analytic sample consisted of N = 374 223 patients from 568 facilities with at least 2 of 3 provider types. We created 3 nonoverlapping cohorts of patients in facilities with at least 2 types of providers serving PCP roles for patients in our cohort: (1) primary analysis—all 3 provider types (N = 153 057 from 139 facilities); (2) secondary analysis—physician and NP only (N = 184 452 from 346 facilities), and (3) secondary analysis—physician and PA only (N = 36 714 from 83 facilities).

### Outcome

The outcome of interest is the type of PCP most frequently seen in FYs 2012-2013. This provider type could be an attending physician, NP, or PA. A patient’s PCP was considered to be the individual to whom the patient had the most primary care visits in FYs 2012-2013. If no PCP could be assigned (i.e., the patient saw multiple providers equally), the patient was excluded from the analysis.

### Explanatory Variables Included in Multivariable Regression Analyses

Our choice of patient-level variables to examine for association with PCP type was informed by the determinants of health model developed by Evans and Stoddart.^41^ In Table 1, we map each of the patient-level variables in our study to the Evans and Stoddart categories and then modify the model by mapping the variables to the concepts of medical and social complexity that are central to our research question. The measures most relevant to medical complexity were age and the Diagnostic Cost Group (DCG) score, while most of the remaining variables measured social complexity. The DCG comorbidity measure was originally designed to predict cost of care but has been validated to measure medical complexity within the VHA population.^42,43^ The algorithm uses demographic and diagnostic information to assign each patient a DCG score, normed so that the average Medicare patient has a score equal to 1.^44^ All patient-level variables were constructed using VHA EHR data from FY 2012.

**Table 1.** Rationale for Patient-Level Variable Selection.

Determinants of health^41^	Variables in our study^a^	Measures of medical complexity	Measures of social complexity
Social environment	Age	+	
	Sex		
	Race		+
	Ethnicity		+
	Marital status		+
	Distance from assigned station		+
Physical environment	Homelessness		+
Prosperity	Copayment status based on poverty^b^		+
Disease			+
Physical disease	VA medical complexity score (DCG)^44^	+	
Mental disease	Psychiatric conditions (non-PTSD and nonmood disorders)		+
	PTSD		+
	Mood disorders		+
	Dementia		+
Behavior	Substance abuse		+
Health and function	Copayment status based on disability		+

*Note.* VA = Veterans Administration; DCG = Diagnostic Cost Group; PTSD = posttraumatic stress disorder.

a The Evans and Stoddart model also includes categories for genetic factors and well-being. Variables representing these categories were not available for use in our study.

b For determination of VA patient copay status, patients are first considered for exemption from copay on the basis of disability. If they do not qualify for exemption based on disability, they are considered for exemption based on poverty. Therefore, the group classified as “low income” variable does not represent low income disabled veterans.

Facility- and state-level contextual variables including multispecialty (vs single-specialty) nature of facilities, rural-urban status, and region have been associated with use of NPs and PAs.^27,32^ The proportion of providers in the facility who were PAs or NPs would also obviously affect the chance of a patient being assigned to NP or PA care. Variables representing these characteristics were examined, as shown in Table 2. The availability of endocrinology referrals within the facility was included as a proxy for facility complexity and multispecialty availability for diabetes care. The proportion of providers who were PAs or NPs and the presence of endocrinology and/or specialty diabetes services at a facility (defined as providing 500 or more such visits in FY 2012) were calculated using our analytic dataset. Facility-level rural-urban status was based on the ZIP code version of the Rural Urban Community Area (RUCA) codes.^45^ Region of facility is designated as assigned by the VHA.

**Table 2.** Characteristics of VHA Patients With Diabetes by Primary Care Provider Type Assigned in Facilities With Physicians, NPs, and PAs.

Category	Physician-assigned provider (n = 113 475)	NP-assigned provider (n = 21 513)	PA-assigned provider (n = 18 069)	Total (N = 153 057)
Patient-level factors				
Male	109 746 (96.71)	20 100 (93.43)	17 600 (97.40)	147 446 (96.33)
Age group				
Less than 40	1068 (0.94)	274 (1.27)	193 (1.07)	1535 (1.00)
40 to less than 65	58 634 (51.67)	11 076 (51.49)	9118 (50.46)	78 828 (51.50)
65 to less than 80	43 190 (38.06)	8178 (38.01)	7055 (39.04)	58 423 (38.17)
80 and above	10 583 (9.33)	1985 (9.23)	1703 (9.42)	14 271 (9.32)
Race				
White	76 326 (67.26)	15 235 (70.82)	13 074 (72.36)	104 635 (68.36)
American Indian	864 (0.76)	206 (0.96)	130 (0.72)	1200 (0.78)
Asian	453 (0.40)	119 (0.55)	51 (0.28)	623 (0.41)
Black	25 664 (22.62)	3950 (18.36)	3268 (18.09)	32 882 (21.48)
Native Hawaiian	1260 (1.11)	209 (0.97)	132 (0.73)	1601 (1.05)
Unknown or Missing	8908 (7.85)	1794 (8.34)	1414 (7.83)	12 116 (7.92)
Hispanic	5870 (5.17)	786 (3.65)	711 (3.93)	7367 (4.81)
Marital status				
Currently married	66 521 (58.62)	12 439 (57.82)	10 951 (60.61)	89 911 (58.74)
Never married	13 532 (11.93)	2556 (11.88)	1880 (10.40)	17 968 (11.74)
Previously married	33 218 (29.27)	6436 (29.92)	5197 (28.76)	44 851 (29.30)
Unknown marital status	204 (0.18)	82 (0.38)	41 (0.23)	327 (0.21)
Homeless at any time during year	2395 (2.11)	517 (2.40)	298 (1.65)	3210 (2.10)
Copay status				
No copay due to disability	62 865 (55.40)	11 520 (53.55)	9674 (53.54)	84 059 (54.92)
No copay due to low income	30 228 (26.64)	5656 (26.29)	4750 (26.29)	40 634 (26.55)
Must pay copay	18 843 (16.61)	3871 (17.99)	3420 (18.93)	26 134 (17.07)
Copay status unknown	1539 (1.36)	466 (2.17)	225 (1.25)	2230 (1.46)
Mental health diagnoses				
Mood disorder	27 424 (24.17)	5372 (24.97)	4233 (23.43)	37 029 (24.19)
Posttraumatic stress disorder	16 106 (14.19)	3169 (14.73)	2494 (13.80)	21 769 (14.22)
Dementia	3669 (3.23)	611 (2.84)	560 (3.10)	4840 (3.16)
Substance abuse	9142 (8.06)	1736 (8.07)	1260 (6.97)	12 138 (7.93)
Other mental health diagnosis	6888 (6.07)	1396 (6.49)	1088 (6.02)	9372 (6.12)
DCG score category				
Less than or equal to 0.5	54 258 (47.81)	10 732 (49.89)	9199 (50.91)	74 189 (48.47)
Greater than 0.5 to 1	18 439 (16.25)	3650 (16.97)	2966 (16.41)	25 055 (16.37)
Greater than 1 to 1.5	15 305 (13.49)	2932 (13.63)	2386 (13.20)	20 623 (13.47)
Greater than 1.5 to 2	8772 (7.73)	1502 (6.98)	1303 (7.21)	11 577 (7.56)
Greater than 2	16 701 (14.72)	2697 (12.54)	2215 (12.26)	21 613 (14.12)
Distance from VHA primary care clinic				
Less than 5 miles	22 697 (20.00)	4885 (22.71)	4070 (22.52)	31 652 (20.68)
5 to less than 25 miles	61 486 (54.18)	10 486 (48.74)	8633 (47.78)	80 605 (52.66)
25 to less than 50 miles	18 766 (16.54)	3903 (18.14)	3440 (19.04)	26 109 (17.06)
50 miles and more	9845 (8.68)	1941 (9.02)	1822 (10.08)	13 608 (8.89)
Missing	681 (0.60)	298 (1.39)	104 (0.58)	1083 (0.71)
Facility-level factors				
Endocrinology referral capacity^a^	81 168 (71.53)	12 596 (58.55)	9748 (53.95)	103 512 (67.63)
Percent of providers in primary care clinic who are PAs				
Lowest tertile	57 330 (50.52)	10 381 (48.25)	2911 (16.11)	70 622 (46.14)
Middle tertile	39 196 (34.54)	6701 (31.15)	6170 (34.15)	52 067 (34.02)
Highest tertile	16 949 (14.94)	4431 (20.60)	8988 (49.74)	30 368 (19.84)
Percent of providers in primary care clinic who are NPs				
Lowest tertile	56 452 (49.75)	2729 (12.69)	7696 (42.59)	66 877 (43.69)
Middle tertile	39 556 (34.86)	8310 (38.63)	7104 (39.32)	54 970 (35.91)
Highest tertile	17 467 (15.39)	10 474 (48.69)	3269 (18.09)	31 210 (20.39)
Rural-urban commuting area status				
Metropolitan area core	99 992 (88.12)	18 798 (87.38)	14 037 (77.69)	132 827 (86.78)
Metropolitan area core—remaining levels	8992 (7.92)	1343 (6.24)	1882 (10.42)	12 217 (7.98)
Micropolitan area core	3948 (3.48)	1208 (5.62)	1810 (10.02)	6966 (4.55)
Small town or rural	543 (0.48)	164 (0.76)	340 (1.88)	1047 (0.68)
State-level factors				
Region				
Northeast	15 259 (13.45)	3420 (15.90)	3500 (19.37)	22 179 (14.49)
West	13 465 (11.87)	4997 (23.23)	2404 (13.30)	20 866 (13.63)
Midwest	33 050 (29.13)	7181 (33.38)	5116 (28.31)	45 347 (29.63)
South	51 701 (45.56)	5915 (27.50)	7049 (39.01)	64 665 (42.25)
Percent of primary care physicians who work with NPs/PAs				
Lowest tertile	61 335 (54.05)	8945 (41.58)	7982 (44.18)	78 262 (51.13)
Middle tertile	28 495 (25.11)	7006 (32.57)	5426 (30.03)	40 927 (26.74)
Highest tertile	23 645 (20.84)	5562 (25.85)	4661 (25.80)	33 868 (22.13)
Physician assistant scope of practice regulations				
Least restrictive	18 390 (16.21)	3762 (17.49)	3221 (17.83)	25 373 (16.58)
Moderately restrictive	21 307 (18.78)	4600 (21.38)	3564 (19.72)	29 471 (19.25)
Most restrictive	73 778 (65.02)	13 151 (61.13)	11 284 (62.45)	98 213 (64.17)
Nurse practitioner scope of practice regulations				
Least restrictive	14 084 (12.41)	5226 (24.29)	2824 (15.63)	22 134 (14.46)
Moderately restrictive	15 546 (13.70)	2905 (13.50)	2385 (13.20)	20 836 (13.61)
Most restrictive	83 845 (73.89)	13 382 (62.20)	12 860 (71.17)	110 087 (71.93)

*Note.* Data for patient-level variables are from the VA electronic health record files. Other data sources are described in the “Methods” section. PCP is assigned as the physician, NP, or PA seen most during FY 2012 and 2013. VA = Veterans Administration; NP = nurse practitioner; PA = physician assistant; DCG = Diagnostic Cost Group; VHA = Veterans Health Administration; PCP = primary care provider; FY = fiscal year.

a Endocrinology referral capacity is defined as either present (endocrinology or other diabetes mellitus specialty clinics provided 500 or more visits to cohort patients in FY 2012) or absent (fewer than 500 visits to cohort patients).

Additional state-level contextual variables were examined. The proportion of primary care physicians within a state who report working with NPs or PAs was obtained from an analysis of the National Ambulatory Medical Care Survey by Hing and Hsiao.^32^ This variable was included to reflect the level of acceptance and uptake of PAs and NPs in the state. NP SOP ratings were obtained from the 2012 Pearson Report, with the most restrictive SOP environment requiring physician involvement for an NP to prescribe, diagnose, or treat, the moderately restrictive environment requiring physician involvement for an NP to prescribe medications, and the least restrictive environment requiring no physician involvement.^46^ PA SOP regulation scores were calculated by the authors using a tabulation of recommended key elements (“licensure” as the regulatory term, full prescriptive authority, SOP and chart cosignature requirements determined at the practice level, adaptable collaboration rules, and no restriction on the number of PAs a physician can supervise) assembled by the American Academy of Physician Assistants, with presence of 3 key elements indicating the most restrictive SOP, 4 elements indicating moderately restrictive SOP, and 5 to 6 elements categorized as the least restrictive SOP.^47^

### Statistical Analysis

We examined the association of PCP types (ie, physicians, NP, or PA) with all patient-level, facility-level, and state-level variables simultaneously using a generalized logit model for the main analysis with all 3 provider types; logistic regression was used for the 2 secondary analyses. In all models, a facility-level random intercept was included to account for clustering within clinics. Covariates were specified a priori and entered into the model simultaneously after examination for collinearity by evaluating frequency cross-tabulations and cluster analysis results using the SAS VARCLUS procedure. All analyses set statistical significance at *P* < .05 and were conducted using SAS 9.4 (Copyright 2013, SAS Institute Inc., Cary, North Carolina).

The study was approved by the Institutional Review Board of the Durham Veterans Affairs Medical Center.

## Results

### Providers and Clinics of VHA Patients With Diabetes Mellitus

Among 5,774,903 VHA patients seen in FY 2012, 830,602 (14.4%) individuals met our diagnostic criteria for pharmacologically treated diabetes mellitus. Among diabetic patients for whom we could assign a nonresident PCP (456,985 patients in 823 facilities), physicians were the most frequent PCP type (78%), followed by NPs (16%) and PAs (6%).Among these 823 facilities, 17% (n = 139) had all 3 types of providers, 42% (n = 346) had only physicians and NPs, and 10% (n = 83) had only physicians and PAs (see Figure 1). Patients in the remaining 255 facilities were excluded from our analytic sample because they only had 1 provider type represented and/or did not have any physicians represented in the data. It is important to remember that these clinics might have employed physicians or other provider types who did not appear in our study because none of their patients met our study inclusion criteria.

### Characteristic of Diabetic Patients in Our Analytic Sample

Our primary analysis was among the subset of clinics that had all 3 provider types (physician, NP, and PA) serving as PCPs for patients. This subset comprised 139 clinics with 153,057 patients, representing 41% of patients in our analytic sample. Among these clinics, physicians were the PCP for 74% of cohort patients, NPs were the PCP for 14%, and PAs were the PCP for 12%. Characteristics of the patients in this subset are shown in Table 2, and characteristics of the remaining 2 subsets in our secondary analyses (patients attending clinics with only physicians and NPs serving as PCPs for cohort patients and patients attending clinics with only physicians and PAs serving as PCPs for cohort patients) are presented in the appendix.

Like the general VHA population, the patients with diabetes who attended clinics with all 3 provider types are predominantly male (96%) and older (47% aged 65 and above) than the general US population. They are medically and socially complex, with relatively high rates of mental health disorders (mood disorders 24%, posttraumatic stress disorder [PTSD] 14%, and substance abuse 8%), homelessness (2%), and exemption from copays due to disability (55%) or due to low income (26%). Compared with patients attending clinics with only 2 provider types serving as PCPs for cohort patients (Appendix A), patients attending clinics with all 3 provider types serving as PCPs for cohort patients are more urban (87%), more likely to be located in the South (42%) and Midwest (30%), and more likely to be African American (21%).

### Factors Associated With PCP Type

In facilities where all 3 provider types cared for cohort patients, after adjustment for all other factors, the odds of having an NP or PA as PCP steadily declined as complexity and age increased (Table 3). For the group with the highest DCG score (>2.0) compared with the group with the lowest (0-0.5), the odds ratio (OR) of having an NP PCP compared with a physician was 0.83 (95% confidence interval [CI]: 0.79-0.88) and for having a PA compared with a physician was also 0.85 (CI: 0.80-0.89). For the oldest age group compared with the youngest, the OR for having an NP PCP was 0.67 (CI: 0.57-0.79) and the OR for having a PA PCP was 0.67 (CI: 0.56-0.79). Similar patterns were found in the clinics with only 2 provider types (Appendix A).

**Table 3.** ORs for Predicting Assignment to a Primary Care Provider Who Is an NP or PA, Compared With Physician Among Facilities With Physician, NP, and PA Provider Types.

Effect and level	OR for assignment to NP	95% CI for assignment to NP	*P* value for assignment to NP	OR for assignment to PA	95% CI for assignment to PA	*P* value for assignment to PA
Patient-level factors						
Male	0.42	0.39-0.45	<.001	1.28	1.14-1.43	<.001
Age group						
Less than 40	Reference	Reference		Reference	Reference	
40 to less than 65	0.74	0.63-0.85	<.001	0.75	0.63-0.88	<.001
65 to less than 80	0.72	0.62-0.83	<.001	0.73	0.62-0.87	<.001
80 and above	0.67	0.57-0.79	<.001	0.67	0.56-0.79	<.001
Race						
White	Reference	Reference		Reference	Reference	
American Indian	1.00	0.84-1.18	.956	1.00	0.82-1.21	.962
Asian	1.04	0.83-1.30	.730	1.05	0.77-1.42	.768
Black	1.01	0.96-1.06	.705	1.09	1.04-1.15	<.001
Native Hawaiian	1.00	0.85-1.18	.979	0.81	0.67-0.98	.033
Unknown or Missing	1.02	0.96-1.09	.431	1.02	0.95-1.09	.620
Hispanic	0.95	0.87-1.04	.273	1.03	0.95-1.13	.449
Marital status						
Currently married	Reference	Reference		Reference	Reference	
Never married	1.02	0.96-1.07	.539	1.02	0.96-1.08	.447
Previously married	1.04	1.00-1.07	.068	1.04	1.00-1.08	.048
Unknown marital status	1.49	1.13-1.97	.005	1.23	0.86-1.76	.254
Homeless at any time during year	1.16	1.04-1.29	.007	1.09	0.95-1.24	.225
Copay status						
Must pay copay	Reference	Reference		Reference	Reference	
No copay due to disability	0.94	0.90-0.98	.008	0.94	0.90-0.99	.013
No copay due to low income	0.96	0.91-1.01	.130	0.96	0.91-1.01	.093
Unknown copay status	1.04	0.89-1.21	.600	0.92	0.77-1.10	.356
Mental health diagnoses						
Mood disorder	1.01	0.96-1.05	.785	0.99	0.95-1.04	.747
PTSD	1.05	1.00-1.10	.076	1.02	0.96-1.07	.538
Dementia	0.89	0.80-0.98	.021	0.99	0.89-1.10	.808
Substance abuse	1.11	1.04-1.18	.001	1.01	0.94-1.08	.827
Other mental health diagnosis	1.19	1.11-1.28	<.001	1.09	1.01-1.17	.035
DCG score category						
Less than or equal to 0.5	Reference	Reference		Reference	Reference	
Greater than 0.5 to 1	1.00	0.95-1.04	.880	0.96	0.92-1.01	.110
Greater than 1 to 1.5	0.95	0.91-1.00	.060	0.95	0.90-1.00	.041
Greater than 1.5 to 2	0.86	0.81-0.92	<.001	0.92	0.86-0.99	.022
Greater than 2	0.83	0.79-0.88	<.001	0.85	0.80-0.89	<.001
Distance from VHA primary care clinic						
Less than 5 miles	Reference	Reference		Reference	Reference	
5 to less than 25 miles	1.00	0.96-1.04	.982	1.04	0.99-1.09	.082
25 to less than 50 miles	1.04	0.99-1.10	.121	1.09	1.03-1.15	.003
50 miles or greater	1.00	0.94-1.07	.982	1.15	1.07-1.23	<.001
Missing	1.72	1.40-2.11	<.001	1.37	1.05-1.79	.021
Facility-level factors						
Endocrinology referral capacity^a^	0.87	0.68-1.12	.284	0.80	0.62-1.04	.097
Percent of providers in the primary care clinic who are PAs						
Lowest tertile	Reference	Reference		Reference	Reference	
Middle tertile	1.05	0.79-1.40	.724	4.40	3.29-5.89	<.001
Highest tertile	1.63	1.20-2.22	.002	18.19	13.32-24.84	<.001
Percent of providers in the primary care clinic who are NPs						
Lowest tertile	Reference	Reference		Reference	Reference	
Middle tertile	6.35	4.65-8.66	<.001	1.14	0.84-1.55	.413
Highest tertile	21.54	15.80-29.38	<.001	1.71	1.26-2.33	<.001
Rural-urban commuting area status						
Metropolitan area core	Reference	Reference		Reference	Reference	
Metropolitan area core—remaining levels	0.97	0.63-1.48	.884	0.90	0.59-1.37	.620
Micropolitan area core	1.07	0.69-1.65	.773	1.10	0.71-1.70	.662
Small town or rural	1.32	0.60-2.88	.492	1.47	0.66-3.29	.345
State-level factors						
Region						
Northeast	Reference	Reference		Reference	Reference	
West	0.72	0.46-1.11	.137	0.60	0.38-0.93	.024
Midwest	0.69	0.48-0.98	.039	0.79	0.55-1.13	.193
South	0.86	0.60-1.22	.389	0.88	0.62-1.26	.483
Percent of primary care physicians who work with NPs/PAs						
Lowest tertile	Reference	Reference		Reference	Reference	
Middle tertile	1.07	0.76-1.53	.687	0.84	0.59-1.19	.330
Highest tertile	1.35	0.91-2.00	.139	1.03	0.69-1.54	.869
Nurse practitioner scope of practice regulations						
Lowest tertile	Reference	Reference		Reference	Reference	
Moderately restrictive	0.75	0.45-1.26	.276	1.05	0.63-1.76	.852
Most restrictive	1.12	0.74-1.71	.592	0.90	0.59-1.38	.623
Physician assistant scope of practice regulations						
Lowest tertile	Reference	Reference		Reference	Reference	
Moderately restrictive	1.20	0.78-1.83	.402	1.13	0.74-1.74	.561
Most restrictive	1.15	0.79-1.66	.460	1.15	0.79-1.67	.457

*Note.* Data for patient-level variables are from the VA electronic health record files. Other data sources are described in the “Methods” section. PCP is assigned as the physician, NP, or PA seen most during FY 2012 and 2013. OR = odds ratio; NP = nurse practitioner; CI = confidence interval; PA = physician assistant; PTSD = posttraumatic stress disorder; DCG = Diagnostic Cost Group; VHA = Veterans Health Administration; PCP = primary care provider; FY = fiscal year.

a Endocrinology referral capacity is defined as either present (endocrinology or other diabetes mellitus specialty clinics provided 500 or more visits to cohort patients in FY 2012) or absent (fewer than 500 visits to cohort patients).

Many of the variables indicating social complexity (race, ethnicity, marital status, not being required to pay a VHA copayment due to low income, rural clinic locations, and diagnosis of a mood disorder, PTSD, or dementia) did not show statistically significant differences in the odds of having a particular provider type after adjustment for the other variables. Among variables that did have statistically significant differences, the differences were relatively small. For example, the odds of a patient seeing either an NP or a PA as his or her PCP were about 6% lower than the odds of seeing a physician for patients exempt from copays due to disability compared with those making copayments (NP OR = 0.94, CI: 0.90-0.98; PA OR = 0.94, CI: 0.90-0.99). Similarly, the odds of a patient seeing an NP as his or her PCP were 15% higher than those of seeing a physician for homeless patients (OR = 1.16, CI: 1.04-1.29) and 11% higher for patients with a substance abuse diagnosis (OR = 1.11, CI: 1.04-1.18). PAs were more likely than physicians to see patients with long commutes to their primary care clinic compared with those who lived within 5 miles (OR = 1.15, CI: 1.07-1.23).

In clinics with all 3 provider types caring for cohort patients, NPs were notably less likely to be the PCP for male patients than were physicians (OR = 0.42, CI: 0.39-0.45) and PAs were more likely than physicians to be the PCP for males (OR = 1.28, CI: 1.14-1.43). This pattern held in the secondary analysis of the subset of clinics that had only NPs and physicians as PCPs (OR for males vs females seeing an NP PCP compared with a physician = 0.46, CI: 0.43-0.49). In clinics without NPs, however, this pattern changed, with PAs much less likely than physicians to be the PCP for men (OR = 0.65, CI: 0.54-0.78; Appendix B).

The largest ORs in our regression models were for the proportions of NPs and PAs in the facilities, an obvious result of the necessity of a provider type being present in order for them to be the PCP for a patient. In our primary analysis, the other facility-level factors we examined (availability of endocrine referrals and rural-urban status) did not show statistically significant differences in the odds of having an NP or PA PCP compared with physicians.

Among the state-level variables, we found a lower odds of having an NP than physician provider in the midwest compared with the northeast (OR = 0.69, CI: 0.48-0.98) and a lower odds of having a PA than physician provider in the west, compared with the northeast (OR = 0.60, CI: 0.38-0.93). Neither the percent of primary care physicians in the state who work with NPs or PAs nor state-level SOP regulations were associated with provider type after controlling for all of the factors in our model.

## Discussion

The VHA uses NPs and PAs extensively as PCPs for patients with chronic diseases such as diabetes, many of whom are medically and socially complex. NPs are used to a greater extent than PAs as PCPs for veterans with diabetes, a trend that mirrors national primary care staffing patterns and might also reflect the larger size of the NP workforce compared with the PA workforce.^48^ We found substantial variation among facilities in provider types used, with many facilities having only NPs and physicians as PCPs for patients in our cohort while others had only PAs and physicians.

The modest magnitude of the differences we found in PCP type assignment is consistent with previous research suggesting that physicians, NPs, and PAs functioning in similar roles in similar environments tend to care for similar types of patients and provide similar types of care.^27,30,49-51^ For example, although we found that the most medically complex VHA primary care patients with diabetes mellitus (DCG score >2.0) had about 15% higher odds of having a physician PCP than an NP or PA, there was considerable overlap of patient complexity scores among provider types. While 15% of physician patients were in the most medically complex group, 12% to 13% of PA and NP patients were also in this most complex category.

After controlling for all other factors, including medical complexity, older patients were more likely to have a physician PCP. It is possible that this is due to components of medical complexity that are not completely captured by the DCG score. Alternative explanations are that they might have had a physician PCP for years, before NPs and PAs were commonly used as PCPs, or that older patients prefer physician care. Our study did not address patient preference, but there is some evidence that older patients may be more inclined to choose a physician over an NP or PA when they are given an option.^52^

Social complexity factors were not consistently associated with a particular PCP type. While some statistically significant differences were observed, the magnitude of such differences was small, indicating that social complexity is not a major driver of PCP type.

Although not related to medical or social complexity directly, the gender differences in PCP type are notable. The VHA has women’s health clinics which serve as primary care clinics for many women veterans.^53^ Due to evidence that outcomes are best when women providers provide primary care to women veterans,^54^ these clinics might have been preferentially staffed by NPs, who are overwhelmingly female (93% female in 2012).^55^ Perhaps NPs are also preferentially hired to staff these clinics due to the traditional strong role of NPs in women’s health. It is not clear why PAs seem to fill the role of PCP for women veterans when NPs are not present, although the PA profession has a larger proportion of female providers (66%)^56^ compared with the physician profession (33%).^57^

Other than some minimal regional associations, state-level factors were not associated with PCP provider type. Our finding that the type of PCP assigned to a patient was not affected by state SOP is consistent with recent findings that SOP regulations were not associated with productivity in the VHA^58^ or with practice patterns and care quality in Community Health Centers.^59^

### Strengths and Limitations

Unlike most previous studies which analyze discrete patient encounters or episodes of care for acute uncomplicated conditions, our study evaluated PCP type for chronic disease care over the course of 2 years. This approach is appropriate as analyzing isolated patient encounters or discrete episodes of care is inherently limited for studying primary care for chronic conditions—an enterprise which is by nature continuous and comprehensive. Our approach is also timely as provision of continuous and comprehensive chronic disease care is a central challenge facing the VHA and US health care system as a whole.^60^ Strengths of our data source include accurate attribution of care of each patient to a particular provider, an attribute missing in many commonly used data sources.^37^

Our study analyzed only face-to-face visits with the PCP, but PACT implementation in the VHA has been associated with large increases in non-face-to-face encounters such as phone and electronic communication.^61^ While it is possible that inclusion of these encounters might have affected our assignment of patients to providers, many of the non-face-to-face encounters are provided by nurses or other clinicians not acting in a PCP role.^61^ In addition, PCMH models such as the PACT model emphasize care by the entire team, while our study focuses only on the presumptive team leader. Finally, our study did not have information on some factors that might affect PCP type assignment, such as patient preferences.^62^

## Conclusions

The question of how to best use physicians, NPs, and PAs in primary care is of key importance in the VHA as well as in other health organizations. This study contributes a crucial first step in addressing this question, by examining factors associated with patient assignment by provider type. We found that more medically complex patients had slightly higher odds of having a physician as compared with an NP or PA PCP, but associations of social complexity factors with provider type were modest and inconsistent. Future work will compare quality and cost outcomes by provider type, controlling for the case mix factors we have identified.

## Appendix

**Appendix A:** Characteristics of VA Patients With Diabetes by Primary Care Provider Type Assigned in Facilities With Either Physicians and NPs or Physicians and PAs Assigned.

Category	Clinics with only physicians and NPs assigned as PCPs			Clinics with only physicians and PAs assigned as PCPs		
	Physician-assigned provider (n = 137 667)	NP-assigned provider (n = 46 785)	Total (N = 184 452)	Physician-assigned provider (n = 28 794)	PA-assigned provider (n = 7920)	Total (N = 36 714)
Patient-level factors						
Male	133 933 (97.3)	44 778 (95.7)	178 711 (96.9)	28 004 (97.3)	7655 (96.7)	35 659 (97.1)
Age group						
Less than 40	1219 (0.89)	450 (0.96)	1669 (0.90)	214 (0.74)	67 (0.85)	281 (0.77)
40 to less than 65	70 337 (51.1)	23 393 (50.0)	93 730 (50.8)	14 235 (49.4)	3743 (47.3)	17 978 (49.0)
65 to less than 80	53 202 (38.6)	18 256 (39.0)	71 458 (38.7)	11 459 (39.8)	3221 (40.7)	14 680 (40.0)
80 and above	12 909 (9.38)	4686 (10.0)	17 595 (9.54)	2886 (10.0)	889 (11.2)	3775 (10.3)
Race						
White	97 508 (70.8)	34 284 (73.3)	131 792 (71.5)	22 222 (77.2)	6393 (80.7)	28 615 (77.9)
American Indian	890 (0.65)	273 (0.58)	1163 (0.63)	192 (0.67)	66 (0.83)	258 (0.70)
Asian	1067 (0.78)	171 (0.37)	1238 (0.67)	48 (0.17)	26 (0.33)	74 (0.20)
Black	24 544 (17.8)	7797 (16.7)	32 341 (17.5)	3826 (13.3)	715 (9.03)	4541 (12.4)
Native Hawaiian	1657 (1.20)	404 (0.86)	2061 (1.12)	295 (1.02)	98 (1.24)	393 (1.07)
Unknown or Missing	12 001 (8.72)	3856 (8.24)	15 857 (8.60)	2211 (7.68)	622 (7.85)	2833 (7.72)
Hispanic	6544 (4.75)	1786 (3.82)	8330 (4.52)	900 (3.13)	210 (2.65)	1110 (3.02)
Marital status						
Currently married	82 405 (59.9)	28 156 (60.2)	110 561 (59.9)	18 452 (64.1)	5113 (64.6)	23 565 (64.2)
Never married	14 795 (10.7)	5174 (11.1)	19 969 (10.8)	2507 (8.71)	643 (8.12)	3150 (8.58)
Previously married	39 968 (29.0)	13 316 (28.5)	53 284 (28.9)	7736 (26.9)	2148 (27.1)	9884 (26.9)
Unknown marital status	499 (0.36)	139 (0.30)	638 (0.35)	99 (0.34)	16 (0.20)	115 (0.31)
Homeless at any time during year	2410 (1.75)	735 (1.57)	3145 (1.71)	279 (0.97)	68 (0.86)	347 (0.95)
Copay status						
No copay due to disability	75 781 (55.0)	24 059 (51.4)	99 840 (54.1)	15 318 (53.2)	3944 (49.8)	19 262 (52.5)
No copay due to low income	35 955 (26.1)	12 448 (26.6)	48 403 (26.2)	7293 (25.3)	1972 (24.9)	9265 (25.2)
Must pay copay	24 068 (17.5)	9580 (20.5)	33 648 (18.2)	5790 (20.1)	1879 (23.7)	7669 (20.9)
Copay status unknown	1863 (1.35)	698 (1.49)	2561 (1.39)	393 (1.36)	125 (1.58)	518 (1.41)
Mental health diagnoses						
Mood disorder	32 842 (23.9)	11 177 (23.9)	44 019 (23.9)	6513 (22.6)	1747 (22.1)	8260 (22.5)
Posttraumatic stress disorder	19 787 (14.4)	6253 (13.4)	26 040 (14.1)	4085 (14.2)	961 (12.1)	5046 (13.7)
Dementia	4317 (3.14)	1254 (2.68)	5571 (3.02)	844 (2.93)	187 (2.36)	1031 (2.81)
Substance abuse	10 765 (7.82)	3556 (7.60)	14 321 (7.76)	1669 (5.80)	416 (5.25)	2085 (5.68)
Other mental health diagnosis	8101 (5.88)	2836 (6.06)	10 937 (5.93)	1665 (5.78)	402 (5.08)	2067 (5.63)
DCG score category						
Less than or equal to 0.5	69 438 (50.4)	24 832 (53.1)	94 270 (51.1)	15 668 (54.4)	4662 (58.9)	20 330 (55.4)
Greater than 0.5 to 1	23 807 (17.3)	8219 (17.6)	32 026 (17.4)	4892 (17.0)	1273 (16.1)	6165 (16.8)
Greater than 1 to 1.5	17 338 (12.6)	5830 (12.5)	23 168 (12.6)	3470 (12.1)	897 (11.3)	4367 (11.9)
Greater than 1.5 to 2	9640 (7.00)	2968 (6.34)	12 608 (6.84)	1846 (6.41)	454 (5.73)	2300 (6.26)
Greater than 2	17 444 (12.7)	4936 (10.6)	22 380 (12.1)	2918 (10.1)	634 (8.01)	3552 (9.67)
Distance from VHA primary care clinic						
Less than 5 miles	33 844 (24.6)	12 386 (26.5)	46 230 (25.1)	6833 (23.7)	2143 (27.1)	8976 (24.4)
5 to less than 25 miles	70 610 (51.3)	23 630 (50.5)	94 240 (51.1)	13 673 (47.5)	3949 (49.9)	17 622 (48.0)
25 to less than 50 miles	21 539 (15.6)	7250 (15.5)	28 789 (15.6)	6374 (22.1)	1448 (18.3)	7822 (21.3)
50 miles and more	10 663 (7.75)	3032 (6.48)	13 695 (7.42)	1747 (6.07)	332 (4.19)	2079 (5.66)
Missing	1011 (0.73)	487 (1.04)	1498 (0.81)	167 (0.58)	48 (0.61)	215 (0.59)
Facility-level factors						
Endocrinology referral capacity^a^	65 407 (47.5)	15 476 (33.1)	80 883 (43.9)	7288 (25.3)	1555 (19.6)	8843 (24.1)
Percent of providers in primary care clinic who are NPs/PAs						
Lowest tertile	77 020 (55.9)	7479 (16.0)	84 499 (45.8)	17 320 (60.2)	1147 (14.5)	18 467 (50.3)
Middle tertile	45 587 (33.1)	18 141 (38.8)	63 728 (34.5)	7561 (26.3)	2972 (37.5)	10 533 (28.7)
Highest tertile	15 060 (10.9)	21 165 (45.2)	36 225 (19.6)	3913 (13.6)	3801 (48.0)	7714 (21.0)
Rural-urban commuting area status						
Metropolitan area core	99 340 (72.2)	31 927 (68.2)	131 267 (71.2)	17 590 (61.1)	3366 (42.5)	20 956 (57.1)
Metropolitan area core—remaining levels	18 647 (13.5)	4361 (9.32)	23 008 (12.5)	6190 (21.5)	2334 (29.5)	8524 (23.2)
Micropolitan area core	14 532 (10.6)	7478 (16.0)	22 010 (11.9)	3755 (13.0)	1711 (21.6)	5466 (14.9)
Small town or rural	5148 (3.74)	3019 (6.45)	8167 (4.43)	1259 (4.37)	509 (6.43)	1768 (4.82)
State-level factors						
Region						
Northeast	21 738 (15.8)	11 576 (24.7)	33 314 (18.1)	4329 (15.0)	2337 (29.5)	6666 (18.2)
West	32 153 (23.4)	9314 (19.9)	41 467 (22.5)	1710 (5.94)	761 (9.61)	2471 (6.73)
Midwest	29 297 (21.3)	12 317 (26.3)	41 614 (22.6)	5411 (18.8)	1997 (25.2)	7408 (20.2)
South	54 479 (39.6)	13 578 (29.0)	68 057 (36.9)	17 344 (60.2)	2825 (35.7)	20 169 (54.9)
Percent of primary care physicians who work with NPs/PAs						
Lowest tertile	81 267 (59.0)	23 057 (49.3)	104 324 (56.6)	14 456 (50.2)	4886 (61.7)	19 342 (52.7)
Middle tertile	38 486 (28.0)	16 108 (34.4)	54 594 (29.6)	5573 (19.4)	1301 (16.4)	6874 (18.7)
Highest tertile	17 914 (13.0)	7620 (16.3)	25 534 (13.8)	8765 (30.4)	1733 (21.9)	10 498 (28.6)
Physician assistant scope of practice regulations						
Least restrictive	20 500 (14.9)	8386 (17.9)	28 886 (15.7)	5785 (20.1)	1112 (14.0)	6897 (18.8)
Moderately restrictive	22 218 (16.1)	9165 (19.6)	31 383 (17.0)	1394 (4.84)	956 (12.1)	2350 (6.40)
Most restrictive	94 949 (69.0)	29 234 (62.5)	124 183 (67.3)	21 615 (75.1)	5852 (73.9)	27 467 (74.8)

*Note.* Data for patient-level variables are from the VA electronic health record files. Other data sources are described in the “Methods” section. PCP is assigned as the physician, NP, or PA seen most during FY 2012 and 2013. VA = Veterans Administration; OR = odds ratio; NP = nurse practitioner; PA = physician assistant; DCG = Diagnostic Cost Group; VHA = Veterans Health Administration; PCP = primary care provider; FY = fiscal year.

a Endocrinology referral capacity is defined as either present (endocrinology or other diabetes mellitus specialty clinics provided 500 or more visits to cohort patients in FY 2012) or absent (fewer than 500 visits to cohort patients).

**Appendix B:** ORs for Predicting Assignment to a Primary Care Provider Who Is an NP or PA, Compared With Physician Among Facilities With Physician and NP or Physician and PA.

Effect and level	Clinics with only physicians and NPs assigned as PCPs				Clinics with only physicians and PAs assigned as PCPs		
	OR for assignment to NP	95% CI for assignment to NP	*P* value for assignment to NP	Blank	OR for assignment to PA	95% CI for assignment to PA	*P* value for assignment to PA
Patient-level factors				1			
Male	0.46	0.43-0.49	<.001	2	0.65	0.54-0.78	<.001
Age group				3			
Less than 40	Reference	Reference		4	Reference	Reference	
40 to less than 65	0.86	0.76-0.98	.021	5	0.77	0.55-1.06	.107
65 to less than 80	0.86	0.75-0.97	.016	6	0.77	0.56-1.07	.123
80 and above	0.85	0.74-0.96	.012	7	0.80	0.57-1.12	.191
Race				8			
White	Reference	Reference		9	Reference	Reference	
American Indian	0.91	0.78-1.06	.232	10	0.88	0.62-1.25	.473
Asian	0.98	0.81-1.18	.820	11	0.89	0.49-1.60	.697
Black	1.06	1.02-1.10	.005	12	0.92	0.83-1.02	.103
Native Hawaiian	0.92	0.81-1.04	.184	13	1.13	0.86-1.48	.379
Unknown or Missing	1.02	0.97-1.06	.496	14	0.99	0.88-1.10	.803
Hispanic	1.07	1.00-1.14	.050	15	1.17	0.97-1.42	.092
Marital status				16			
Currently married	Reference	Reference		17	Reference	Reference	
Never married	1.02	0.98-1.07	.270	18	0.93	0.84-1.04	.201
Previously married	0.99	0.96-1.01	.296	19	1.02	0.95-1.09	.651
Unknown marital status	0.87	0.70-1.08	.204	20	0.60	0.33-1.07	.084
Homeless at any time during year	1.07	0.97-1.18	.155	21	1.17	0.86-1.60	.323
Copay status				22			
Must pay copay	Reference	Reference		23	Reference	Reference	
No copay due to disability	0.91	0.88-0.94	<.001	24	0.97	0.90-1.05	.420
No copay due to low income	0.97	0.93-1.01	.098	25	0.94	0.87-1.03	.171
Copay status unknown	0.95	0.85-1.07	.394	26	1.15	0.87-1.52	.330
Mental health diagnoses				27			
Mood disorder	1.06	1.03-1.09	<.001	28	1.01	0.94-1.10	.707
PTSD	0.97	0.93-1.01	.122	29	0.95	0.86-1.04	.267
Dementia	0.86	0.80-0.93	<.001	30	0.86	0.71-1.04	.115
Substance abuse	1.07	1.02-1.12	.010	31	1.05	0.92-1.19	.506
Other mental health diagnosis	1.09	1.03-1.15	.002	32	0.91	0.79-1.04	.158
DCG score category				33			
Less than or equal to 0.5	Reference	Reference		34	Reference	Reference	
Greater than 0.5 to 1	0.99	0.95-1.02	.460	35	0.88	0.81-0.95	.002
Greater than 1 to 1.5	0.97	0.93-1.01	.107	36	0.92	0.84-1.01	.088
Greater than 1.5 to 2	0.87	0.83-0.91	<.001	37	0.83	0.73-0.94	.003
Greater than 2	0.84	0.81-0.88	<.001	38	0.80	0.72-0.89	<.001
Distance from VHA primary care clinic				39			
Less than 5 miles	Reference	Reference		40	Reference	Reference	
5 to less than 25 miles	1.03	1.00-1.06	.060	41	0.99	0.93-1.07	.866
25 to less than 50 miles	1.05	1.01-1.09	.023	42	0.95	0.87-1.04	.251
50 miles or greater	1.05	0.99-1.11	.094	43	0.95	0.82-1.10	.513
Missing	0.88	0.74-1.06	.177	44	0.52	0.33-0.83	.005
Facility-level factors				45			
Endocrinology referral capacity^a^	0.92	0.72-1.18	.520	46	0.96	0.48-1.93	.916
Percent of providers in the primary care clinic who are NPs/PAs				47			
Lowest tertile	Reference	Reference		48	Reference	Reference	
Middle tertile	5.36	4.28-6.71	<.001	49	7.80	4.89-12.45	<.001
Highest tertile	24.37	19.26-30.85	<.001	50	23.88	14.56-39.18	<.001
Rural-urban commuting area status				51			
Metropolitan area core	Reference	Reference		52	Reference	Reference	
Metropolitan area core—remaining levels	1.09	0.83-1.43	.522	53	1.36	0.78-2.37	.274
Micropolitan area core	1.12	0.89-1.42	.329	54	1.21	0.76-1.95	.423
Small town or rural	1.43	1.03-1.98	.033	55	4.30	1.87-9.86	<.001
State-level factors				56			
Region				57			
Northeast	Reference	Reference		58	Reference	Reference	
West	0.81	0.59-1.10	.171	59	1.03	0.50-2.14	.927
Midwest	1.21	0.91-1.62	.182	60	1.20	0.69-2.11	.520
South	0.80	0.62-1.04	.097	61	0.66	0.40-1.08	.097
Percent of primary care physicians who work with NPs/PAs				62			
Lowest tertile	Reference	Reference		63	Reference	Reference	
Middle tertile	0.94	0.75-1.18	.592	64	0.43	0.26-0.73	.002
Highest tertile	0.94	0.72-1.24	.686	65	0.65	0.37-1.15	.137
Physician assistant scope of practice regulations				66			
Least restrictive	Reference	Reference		67	Reference	Reference	
Moderately restrictive	1.25	0.89-1.77	.199	68	0.54	0.25-1.17	.117
Most restrictive	0.93	0.69-1.25	.621	69	0.65	0.37-1.17	.149

*Note.* Data for patient-level variables are from the VA electronic health record files. Other data sources are described in the “Methods” section. PCP is assigned as the physician, NP, or PA seen most during FY 2012 and 2013. OR = odds ratio; NP = nurse practitioner; CI = confidence interval; PA = physician assistant; PTSD = posttraumatic stress disorder; DCG = Diagnostic Cost Group; VHA = Veterans Health Administration; PCP = primary care provider.

a Endocrinology referral capacity is defined as either present (endocrinology or other diabetes mellitus specialty clinics provided 500 or more visits to cohort patients in FY 2012) or absent (fewer than 500 visits to cohort patients).
